# Identifying Chinese Herbal Medicine Network for Endometriosis: Implications from a Population-Based Database in Taiwan

**DOI:** 10.1155/2017/7501015

**Published:** 2017-06-27

**Authors:** Pei-Ju Tsai, Yi-Hsuan Lin, Jiun-Liang Chen, Sien-Hung Yang, Yu-Chun Chen, Hsing-Yu Chen

**Affiliations:** ^1^Division of Chinese Internal Medicine, Center for Traditional Chinese Medicine, Chang Gung Memorial Hospital, Taoyuan, Taiwan; ^2^School of Traditional Chinese Medicine, College of Medicine, Chang Gung University, Taoyuan, Taiwan; ^3^Graduate Institute of Clinical Medical Sciences, College of Medicine, Chang Gung University, Taoyuan, Taiwan; ^4^School of Medicine, Faculty of Medicine, National Yang-Ming University, Taipei, Taiwan; ^5^Department of Mathematics, University of Toronto, Toronto, ON, Canada

## Abstract

**Background:**

Endometriosis is a common but bothersome gynecological disease, and Chinese herbal medicine (CHM) is used for treating endometriosis. The aim of this study is to explore CHM network and core treatments for endometriosis by analyzing nationwide CHM prescription database.

**Methods:**

From 1998 to 2013, the CHM prescriptions made primarily for endometriosis among women diagnosed with endometriosis (ICD-9-CM code: 671) by gynecologists during their reproductive age were collected. CHM network analysis was then carried out by using association rule mining and social network analysis.

**Results:**

A total of 12,986 CHM prescriptions made for endometriosis were analyzed. There were 556 kinds of CHM ever used, and, in average, each prescription was composed of 6.2 CHMs. Gui-Zhi-Fu-Ling-Wan (GZFLW) was used most frequently, followed by* Cyperus rotundus* (28.1% and 18.8% of all prescriptions, resp.). Additionally, the combination of* Cyperus rotundus* with GZFLW (8.0%) was the most frequently used combination of two CHMs. CHM network showed that GZFLW was the core CHM for endometriosis and graphically demonstrated the extensive coverage of TCM syndromes and pathogenesis of endometriosis.

**Conclusions:**

CHM network provides graphical demonstration and summary of commonly used CHMs for endometriosis, and further studies are warranted based on these findings.

## 1. Introduction

Endometriosis, defined as the presence of endometrial implants outside of the endometrial cavity, is a common but rather irritating menstrual disorder for women [1, 2]. The prevalence reported was as high as 10% among women during their reproductive ages and may account for 50% of infertility women [1, 2]. Although endometriosis is not a fatal disease and most patients remain asymptomatic during entire life, the medical undemands still exist due to the distressing and debilitating symptoms or complications, such as pelvic pain, severe dysmenorrhea, dyspareunia, and infertility [3]. Conservative treatment including nonsteroidal anti-inflammatory drugs, oral contraceptives, danazol, medroxyprogesterone acetate, gonadotropin-releasing hormone agonists, and aromatase inhibitors are frequently used for symptom relief [1, 4]. On the other hand, surgical resection and nerve transection may be considered as definitive therapy if no desire of pregnancy and conservative medical therapy fails [1, 4]. Nevertheless, medical interventions hardly improve fertility, diminish endometrioma, or treat complications of deep endometriosis such as ureteral obstruction. Also, hormonal therapy may lead to abnormal uterine bleeding, weight gain, mood change, bone loss, and so on. Therefore, looking for other ways for treatment of endometriosis with better efficacy or less adverse effects is important [5].

Traditional Chinese medicine (TCM) is one of the most commonly used complement therapies to western medicine managements in Taiwan, including Chinese herbal medicine (CHM), acupuncture, moxibustion, cupping, and manual therapy, and these treatments could be used separately or cooperatively based on TCM doctors' decision [6–8]. CHMs and acupuncture are predominantly used to treat or prevent other conditions associated with chronic or recurring pain, and TCM doctors often try to treat underlying diseases and the associated symptoms at the same time [9, 10]. As a disease with complicated pathogenesis, endometriosis patients often seek CHM for curing primary lesions or controlling symptoms [11, 12]. However, the CHM prescriptions for endometriosis are often so complicated that it is difficult to realize the core CHMs, CHM combinations patterns, and the indications for CHM, especially when TCM doctors frequently combine several CHMs in one prescription for menstrual disorders [13–15].

The aim of this study is to identify a CHM network for endometriosis by analyzing a nationwide CHM prescription database in Taiwan. It is difficult to analyze multiple CHMs used in a huge prescription database by using conventional statistical methods. CHM network analysis is proven to be a good tool to demonstrate the CHM prescription patterns and explore the core CHMs, as well as important CHM combinations [16]. These findings are helpful in overviewing the TCM principle for treating endometriosis and making recommendations to choose proper candidates for further studies.

## 2. Materials and Methods

### 2.1. Data Source

To perform a nationwide research about CHM prescriptions, the National Health Insurance Research Database (NHIRD) was used as the data source in this study. This routinely recorded clinical database comprises all the medical records, including managements in outpatient services, hospitalization medical utilization, and even drop-off prescriptions in the pharmacies, reimbursed by the National Health Insurance (NHI) program since 1995. Extensive coverage of Taiwanese population, over than 99% [17], with interventions done by nearly all subspecialty doctors, including western medicine doctors, TCM doctors, and dentists, makes the NHIRD quite unique when conducting cross-sectional, cohort, and prescription analysis studies [17–20]. This feature is especially important for TCM-related studies, since Taiwan is the handful country that officially recognizes TCM as a potential complement therapy and thus reimburses all TCM therapies, such as Chinese herbal medicine (CHM), acupuncture, moxibustion, and manual therapy. Therefore, this database becomes a valuable source to analyze CHM prescriptions, and the high coverage of entire population makes the results of analysis turn into a sort of consensus among nearly all TCM doctors in Taiwan. Aside from medications, this database contains detailed patients' information, for example, gender, insured level, living places, reasons for outpatients and inpatients' services, expense of each visit and admission, examinations, and expenses. The International Classification of Diseases, Ninth Revision, Clinical Modification (ICD-9-CM) is used to present the reasons for medical use, while the first ICD-9-CM diagnosis code is required to be the primary reason of every visit or admission. The validity and reliability of using ICD-9-CM code as reasons of medical use were reported in previous report [21, 22]. Moreover, since this database is public to all clinical researchers in Taiwan, the patients' identities are well encrypted to protect privacy so that it is impossible to recognize the real identity of patients.

### 2.2. Study Design

To precisely identify endometriosis patients, women who had at least once diagnosis with endometriosis (ICD-9-CM code: 671) by gynecologists from 1998 to 2013 were included in this study and all prescribed CHM prescriptions were extracted from a 1 million representative dataset. This representative dataset was randomly sampled from the entire database, and no statistical differences in gender and age distribution were reported between sampled and entire databases [13]. Further, age restriction, between 20 and 50 years, was used to increase the accuracy of diagnosis. Although there is no definite prevalent age for endometriosis, women during their reproductive ages complained about endometriosis-related symptoms and complications most commonly [23]. Moreover, early onset of dysmenorrhea and dysmenorrhea may be related to primary dysmenorrhea rather than endometriosis, the most common cause to secondary dysmenorrhea, and the possibility of misdiagnosis is lowering when aging [14, 24]. Consequently, the age limitation between 20 and 50 years was then set to select the most appropriate subjects for prescription analysis. After recognizing the endometriosis patients, all CHM prescriptions made for these patients were all retrieved from the database. To ensure that the CHM prescriptions were made for endometriosis, only the prescriptions given with first diagnosis with endometriosis were collected for the final analysis. Visits with managements of acupuncture, moxibustion, and manual therapies were excluded.

### 2.3. Chinese Herbal Medicine (CHM) Prescription in Taiwan

There are two kinds of CHM reimbursed by the NHI, herbal formula (HF) and single herb (SH). SH is the extract or crude powder of a part of herbal plants, insects, animals, and even minerals and is made in accordance with the process methods mentioned in the ancient classics. On the other hand, HF is composed of more than one kind of SH with the same proportion recorded in the TCM classics and is premixed in the pharmaceutical factory before marketing. There were more than 600 kinds of SH and HF available for TCM doctors to choose freely, and all SH and HF were manufactured by the Good Manufacturing Practice pharmaceutical factory with strict regulation about concentration of heavy metal and pesticide.

### 2.4. Bias Assessment

Although the case number may decrease promptly, the use of age restriction on endometriosis diagnosed by the gynecologists and the major diagnosis-only TCM users are helpful to minimize the potential detection bias by using ICD-9-CM as the only inclusion criteria for endometriosis patients, as mentioned in previous studies [14, 25]. Additionally, using this nationwide database can reduce the selection bias due to the high coverage of the NHI and hospital shopping may increase the risk of the selection bias by using only hospital-based database [26]. Furthermore, exclusion of ambulatory visits with acupuncture, moxibustion, and manual therapy is also helpful in avoiding the potential confounding bias on CHM prescription caused by other TCM therapies.

### 2.5. Ethical Consideration

The detailed protocol of this work is reviewed and exempted by the Institutional Review Board of the Chang Gung Memorial Foundation (number 201700066B1), since the individual's identity is well encrypted in the database, and the privacy information cannot be easily traced or recognized.

### 2.6. Statistics Analysis

Association rule mining (ARM) and social network analysis (SNA) were incorporated into analyzing CHM prescriptions and establishing CHM network for endometriosis. The detailed algorithm and data process were described in detail in our previous work [14, 16]. Briefly, by using ARM at first, commonly used combinations of CHM could be identified once three preset criteria were fulfilled: higher-than-threshold support, confidence, and lift factors [16, 27]. Higher support of certain CHM or CHM combinations meant higher prevalence among all possible combinations. Besides, higher values of confidence and lift factors symbolize the stronger connections between CHMs [16]. The decision about thresholds of these three factors mainly depended on clinical experiences, and these values were set to 30% for confidence, 1% for support, and 1 for lift, which were the same as our previous report about primary dysmenorrhea [14]. Furthermore, all commonly used CHM combinations were then processed by the SNA to study the connections between all CHM candidates, define the clusters of CHMs by each of CHM's connection features, and, more importantly, graphically demonstrate the CHM network for endometriosis. Thereafter, core CHMs and important connections to other CHMs with great potentials for endometriosis could also be explored by CHM network. The “arule” package in the “R” software and the software “NodeXL” were used to carry out the statistic calculations in this work.

## 3. Results

### 3.1. Features of CHM Users for Endometriosis

From 2008 to 2013, a total of 33,235 endometriosis patients were recognized by gynecologists, and 31,650 patients ever used TCM at least once for any reason. Among all TCM users, 10,380 patients ever made 81,278 ambulatory visits for dysmenorrhea, dyspareunia, infertility, and pelvic pain, in addition to endometriosis. Finally, a total of 1,736 endometriosis patients made 13,649 visits for endometriosis as their major complaint, and more than 95% visits (12,986 visits) were made for CHM treatments.

### 3.2. CHM Commonly Used for Endometriosis

**Figure 1:**
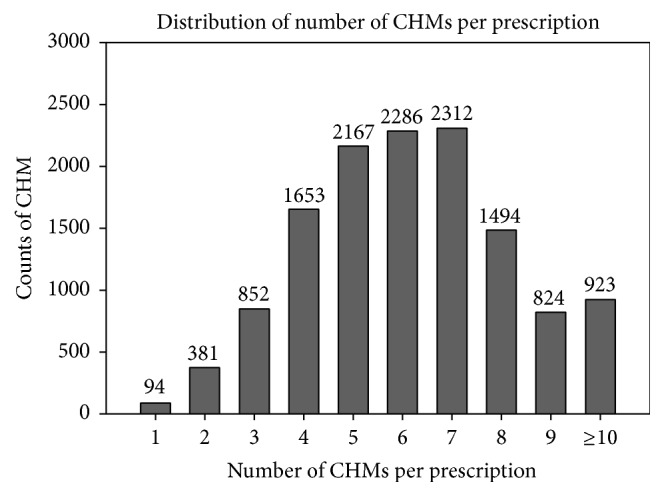
Distribution of Chinese herbal medicines (CHMs) number per prescription.

There were 556 kinds of CHMs ever used for endometriosis patients, and there were 6.2 CHMs prescribed in one prescription in average. Most prescriptions were composed of 7 CHMs (17.8% of all prescriptions), and even more than 7% of prescriptions contained at least 10 kinds of CHMs (Figure 1). Among all HF, Gui-Zhi-Fu-Ling-Wan (GZFLW) was used most frequently, 28.1% of all prescriptions, and it was also the most commonly used CHM for endometriosis (Table 1). Further, Jia-Wei-Xiao-Yao-San and Dang-Gui-Shao-Yao-San were the second and third commonly used HF, 17.2% and 15.0% of all prescriptions, respectively. On the other hand,* Cyperus rotundus*, xiang fu in Chinese, was the most commonly used SH (18.8% of all prescriptions), followed by* Corydalis yanhusuo* (16.9%) and* Leonurus heterophyllus* (11.6%) (Table 2). The average dosage of HF was around 4 gm/day, which was 3-4 times higher than SH (1–1.5 gm/day) (Tables 1 and 2).

### 3.3. Combinations of CHMs Commonly Used for Endometriosis

Table 3 showed the top 10 commonly used two CHMs in combination.* Cyperus rotundus* combined with GZFLW was used most frequently, 8% of all prescriptions, followed by* Sparganium stoloniferum* combined with* Curcuma phaelculis* and* Corydalis yanhusuo* combined with* Cyperus rotundus* (6.5% and 5.9%, resp.). Additionally, the commonly used 3 CHMs in combination were listed in Table 4; the combination of* Sparganium stoloniferum*,* Curcuma phaelculis*, and GZFLW was most prevalent among all possible combinations of CHMs. From the contents of 2 and 3 CHMs in combination, most of them had GZFLW, which implied the crucial role of GZFLW for endometriosis.

### 3.4. CHM Network for Endometriosis

**Figure 2:**
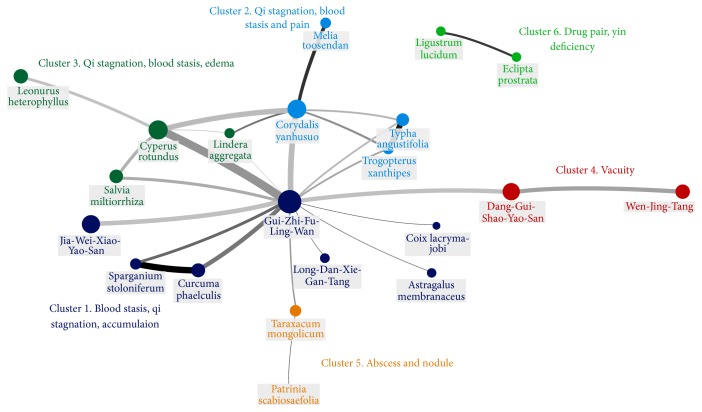
Chinese herbal medicine (CHM) network for endometriosis with clusters of different TCM indications as labeled. Different colors represent different clusters of CHM. Larger circle means higher frequency of using CHM, darker connection lines indicate stronger association, and thicker connection lines mean higher frequency of CHM combinations being used.

Figure 2 graphically demonstrated the CHM network for endometriosis, which was made from the top 30 significant CHMs combinations. Six clusters can be defined automatically by SNA based on the features of every connection between CHMs. Each cluster had its own specific TCM indications by summarizing the effects of within-group CHMs. Cluster 1 was the largest and most prevalent group of CHMs, with GZFLW as its core CHM of this group, and GZFLW was also the core treatment for endometriosis due to its high prevalence and connections to other commonly used CHMs. Other clusters of CHMs seemed to be combined with GZFLW to achieve the therapeutic goal.* Corydalis yanhusuo* in cluster 2 may strengthen the analgesic effect by enhancing the effects on TCM syndrome qi stagnation and blood stasis provided by GZFLW, as well as other CHMs within cluster 2, such as strongly combined* Typha angustifolia* and* Trogopterus xanthipes*.* Cyperus rotundus* in cluster 3 may increase the efficacy on blood stasis, qi stagnation, and edema, while Dang-Gui-Shao-Yao-San in cluster 4 may relieve the vacuity syndrome not provided by GZFLW. The combination of* Eclipta prostrata* with* Ligustrum lucidum* was isolated from other clusters and they formed strong connections between each other.

Additionally, to understand the role of core CHMs for endometriosis, the potential pharmacologic mechanisms were reviewed and summarized in Table 5 (last assessed date: 29/12/2016). Extensive coverage of the pathogenesis of endometriosis could be found by incorporating potential pharmacologic effects into CHM network, such as inducing anti-inflammation, antioxidation, analgesia, correction of luteal effect, and induction of endometrial tissue apoptosis.

## 4. Discussion

This is the first report about graphical and network-based demonstration of CHM therapy for endometriosis to the best of our knowledge. CHM network analysis based on important CHMs combinations is a time-saving and practical method to explore the core CHMs and important CHMs combinations in large-scale clinical prescription database [16]. Since the CHM prescriptions are usually composed of several kinds of CHMs, like 6-7 kinds of CHMs in this study, the CHM network analysis can better demonstrate the relationships between CHMs in a prescription compared with simply ranking commonly used single CHMs. Six clusters of CHMs could be found with their own core CHMs and indications, except cluster six, which is an individual drug pair. Cluster 1, indicated for TCM syndrome “blood stasis, qi stagnation, and accumulation,” is the largest cluster with highest prevalence among all CHM combinations. This finding corresponds to the most prevalent TCM syndrome of endometriosis patients in Taiwan [53].

Moreover, the demonstration on relations between clusters of CHMs is helpful in recognizing which CHMs were prescribed to extend the coverage of TCM syndromes as an adjuvant therapy. Those are quite important references to TCM doctors in clinical practice. Although qi stagnation with blood stasis is the most common TCM syndrome among endometriosis patients, which could be treated mainly by GZFLW, TCM doctors usually combine other CHMs to cover other associated minor TCM syndromes such as vacuity syndrome or to increase the efficacy on qi stagnation. Additionally, the combination of cluster 2 and GZFLW may improve the effectiveness on more serious qi stagnation and blood stasis, which may precipitate painful sensation among endometriosis patients and may not be fully treated by GZFLW alone. On the other hand, the hormone imbalance, which is like vacuity syndrome in TCM theory and is not treated by GZFLW, can be corrected by adding the CHMs within cluster 4, such as Dang-Gui-Shao-Yao-San and Wen-Jing-Tang. This diagnosis-treatment process is known as “bian-zheng-lun-zhi” in TCM theory, which means making up prescriptions according to each patient's TCM syndrome, “zheng” in Chinese [10]. The main part of prescription, sovereign medicinal in TCM theory or the core CHM in this study, is used for the primary TCM syndrome and other CHMs are used as mutual reinforcement or assistance to the main part of prescription.

The graphic demonstration can facilitate exploring the potential core formula in addition to combination patterns between CHMs, and the core formulas are usually the center of a cluster, in which other CHMs may be combined with the core formula to achieve effectiveness. GZFLW seems to be the core CHM for endometriosis, as the center of CHM network, which is almost contained in all important CHM combinations as well as the most prevalent CHMs for endometriosis (Tables 3 and 4). Aside from the well coverage of TCM syndrome for endometriosis, GZFLW may reduce the inflammatory response by suppressing expression of tumor necrosis factor-alpha (TNF-*α*) [28] and reduce the size of endometrial tissues by inducing endometrial cell apoptosis and modulating the aberrant immune response in endometrial cells [31–33]. These are all important principles to manage endometriosis in clinical setting. Additionally, the combination use with GZFLW and* Cyperus rotundus* (the core CHM of cluster for blood stasis, qi stagnation, and edema) may strengthen the apoptosis effect and anti-inflammation effect of GZFLW and may add antioxidation effect to GZFLW as a complement therapy [39–41]. Furthermore, GZFLW and* Cyperus rotundus* are found most commonly among CHM users with reduced surgery rate in a retrospective cohort [54]. In addition, combining* Corydalis yanhusuo* with GZFLW, which comprise the 4th commonest two CHMs in combination, may reinforce the analgesic effect of GZFLW by enhancing the dopamine D1-related pathway and reducing endometrial tissues [42–44]. Furthermore, Dang-Gui-Shao-Yao-San and Wen-Jing-Tang in the 4th cluster both prescribed for vacuity syndrome may be used as important adjuvant CHMs to correct the luteal defect among women, which may account for infertility among endometriosis patients but are uncovered by GZFLW alone [36, 38]. Since menstrual pain and infertility are common complications to endometriosis, the adjuvant effects of other CHMs to GZFLW may explain why these combinations are frequently used by TCM doctors.

Drug pairs are also important findings of the CHM network (cluster 6 in Figure 2). These couplet medicines are combined to increase each other's therapeutic effects with strong relations and may have no direct or significant connections to core CHMs. Therefore, these drug pairs could be used in diseases other than endometriosis if similar TCM indication is found.* Eclipta prostrata* with* Ligustrum lucidum* is an interesting drug pair commonly used for menopausal syndrome due to its potential estrogenic effect [15, 55]. Therefore, it is important to evaluate the role of this drug pair in treating endometriosis, and the exploration of the mechanisms of this drug pair may provide new viewpoint for endometriosis.

The network demonstration of CHMs for endometriosis reveals the relationships between commonly used CHMs, and these relationships can further explain the rationale of using these CHMs on both TCM theories and pharmacological viewpoints. The considerable discrepancy between commonly used CHMs and previously studied CHMs granted further researches about CHMs for endometriosis, since only few CHMs were ever researched for endometriosis (Table 5). However, there are still some limitations for this study. First, only CHMs reimbursed by the NHIRD were included in the prescription analysis in this study, and the local folk medicine may be omitted in this condition. However, this would not greatly influence the further application of the results of this study, since only the reimbursed CHMs have strict regulation on production and identification and are ready to be used whether in experiments or in the clinical practice. Second, the efficacy of core treatments is not evaluated in this study, since it is not requested to record the symptom severity of endometriosis in the medical records. Instead, we extensively searched the literature about potential mechanisms about the important CHMs and CHMs combinations found in the clinical database. These findings would demonstrate the known and unknown aspects of using CHMs and then facilitate further studies. Third, the exclusion of prescriptions associated with acupuncture and moxibustion as well as prescriptions not mainly used for endometriosis may cause considerable decrease in prescriptions number, but it is helpful and necessary to focus on prescription made for endometriosis by relieving potential confounding bias.

## 5. Conclusion

CHM network analysis on large-scale, nationwide prescription database can disclose the core CHMs and important CHMs combinations, as well as the principle of TCM therapies for endometriosis. The graphical demonstration and summary of CHMs for endometriosis clarify CHM for endometriosis and choosing future study candidates.

## Figures and Tables

**Table 1:** The top 5 most commonly used herbal formulas (HF) for endometriosis.

Rank	Name	Ingredients	TCM indications	Prevalence (%)	Dose (gm/day)	Duration (days/visit)
1	Gui-Zhi-Fu-Ling-Wan (GZFLW)	*Cinnamomum cassia, Poria cocos, Paeonia lactiflora or Paeonia veitchii, Paeonia suffruticosa, Prunus persica*	Blood stasis in the uterus	28.1%	4.14	12.7

2	Jia-Wei-Xiao-Yao-San	*Paeoniae alba, Bupleurum chinense, Atractylodis macrocephala, Poria cocos, Angelica sinensis, Mentha haplocalyx, Glycyrrhiza uralensis, Zingiberis Officinalis Recens, Paeonia suffruticosa radicis, Gardenia jasminoides*	Liver Qi stagnation and spleen deficiency	17.2%	4.24	11.6

3	Dang-Gui-Shao-Yao-San	*Angelica sinensis, Ligustici Chuanxiong, Paeoniae alba, Atractylodis macrocephala, Poria cocos, Alisma plantago-aquatica*	Liver blood deficiency and disharmony of liver and spleen	15.0%	4.42	10.8

4	Shao-Fu-Zhu-Yu-Tang	*Foeniculum vulgare, Zingiberis Recens, Cinnamomum cassia, Angelica sinensis, Ligustici chuanxiong, Paeoniae Rubra*	Qi stagnation and blood stasis	11.9%	4.35	10.0

5	Wen-Jing-Tang	*Evodia rutaecarpa, Cinnamomum cassia, Angelica sinensis, Paeoniae alba, Ligustici Chuanxiong, Panax ginseng, Glycyrrhiza uralensis, Equus asinus, Ophiopogon japonicus, Pinellia ternata, Zingiberis Recens*	Deficient cold and blood stasis	11.1%	4.00	11.6

**Table 2:** The top 10 most commonly used single herbs (SH) for endometriosis.

Rank	Name	TCM indications	Prevalence (%)	Dose (gm/day)	Duration (days/visit)
1	*Cyperus rotundus*	Liver Qi stagnation	18.8%	1.05	11.9
2	*Corydalis yanhusuo*	Qi stagnation and blood stasis	16.9%	1.22	10.6
3	*Leonurus heterophyllus*	Blood stasis	11.6%	1.28	9.7
4	*Curcuma phaelculis*	Qi stagnation and blood stasis	10.3%	1.53	12.4
5	*Salvia miltiorrhiza*	Blood stasis	9.7%	1.23	10.1
6	*Typha angustifolia*	Blood stasis	8.6%	1.24	12.1
7	*Trogopterus xanthipes*	Blood stasis	7.3%	1.06	11.3
8	*Taraxacum mongolicum*	Heat toxin	6.9%	1.63	15.4
9	*Cuscuta chinensis*	Kidney deficiency	6.8%	1.21	10.9
10	*Sparganium stoloniferum*	Qi stagnation and blood stasis	6.8%	1.55	11.3

**Table 3:** The top 10 commonly used two combined Chinese herbal medicines (CHMs) for endometriosis.

Rank	CHM A	CHM B	Prevalence (%)	Confidence	Lift
1	*Cyperus rotundus*	GZFLW	8.0%	40.6	1.4
2	*Sparganium stoloniferum*	*Curcuma phaelculis*	6.5%	91.7	8.5
3	*Corydalis yanhusuo*	*Cyperus rotundus*	5.9%	33.0	1.7
4	*Corydalis yanhusuo*	GZFLW	5.8%	32.5	1.1
5	*Typha angustifolia*	*Trogopterus xanthipes*	5.7%	63.8	8.3
6	Jia-Wei-Xiao-Yao-San	GZFLW	5.7%	31.3	1.1
7	*Curcuma phaelculis*	GZFLW	5.1%	47.1	1.6
8	Dang-Gui-Shao-Yao-San	GZFLW	5.0%	31.9	1.1
9	Wen-Jing-Tang	Dang-Gui-Shao-Yao-San	4.4%	37.6	2.4
10	*Melia toosendan*	*Corydalis yanhusuo*	4.1%	69.2	3.9

GZFLW: Gui-Zhi-Fu-Ling-Wan.

**Table 4:** The top 5 commonly used three combined Chinese herbal medicines (CHMs) for endometriosis.

Rank	CHM A	CHM B	CHM C	Prevalence (%)	Confidence	Lift
1	*Sparganium stoloniferum*	*Curcuma phaelculis*	GZFLW	3.4%	52.5	1.8
2	*Cyperus rotundus*	*Corydalis yanhusuo*	GZFLW	2.5%	42.5	1.4
3	*Typha angustifolia*	*Trogopterus xanthipes*	*Corydalis yanhusuo*	2.3%	40.6	2.3
4	*Typha angustifolia*	*Trogopterus xanthipes*	GZFLW	2.1%	36.3	1.2
5	Jia-Wei-Xiao-Yao-San	*Cyperus rotundus*	GZFLW	2.0%	38.8	1.3

GZFLW: Gui-Zhi-Fu-Ling-Wan.

**Table 5:** Potential mechanisms of commonly used CHM for endometriosis.

CHM	Possible mechanisms
Herbal formula (HF)	
Gui-Zhi-Fu-Ling-Wan (GZFLW)	Anti-inflammation by suppressing TNF-*α* in the endothelial cell culture [28] Suppressing anti-endometrial IgM antibody [29, 30] Reducing endometrial tissue size in rat model by increasing CD4 (+) cells and natural killer cells [31] Attenuation of endometriosis lesions by inducing cell apoptosis [32, 33]

Jia-Wei-Xiao-Yao-San	Anti-inflammation by suppressing mitogen-activated protein kinases and nuclear transcription factor kappa B pathway in RAW 264.7 macrophages [34]

Dang-Gui-Shao-Yao-San	Anti-inflammation by decreasing cyclooxygenases-2 message RNA transcription and production of prostaglandin F2*α* in endometrial epithelial cells [35] Correction of luteal phase defect by increasing progesterone from rat ovarian follicles [36]

Shao-Fu-Zhu-Yu-Tang	Anti-inflammation by inhibiting expression of IL-1*β*, IL-2, IL-10, and IL-12 in primary dysmenorrhea mouse model [37] Recovery from hormonal imbalance in blood stasis rat model [37]

Wen-Jing-Tang	Correction of luteal phase defect among patients [38]

Single herb (SH)	
*Cyperus rotundus*	Antioxidation in rat model [39] Antioxidation and induction of apoptosis in leukemic (K562 and L1210) cell lines [40] Anti-inflammation by decreasing IL-1*β* activity in THP-1 cells [41]

*Corydalis yanhusuo*	Analgesia by enhancing dopamine D1 receptor-mediated pathway [42, 43] Induction of apoptosis by activating P38 and JNK pathway in A549 cells [44]

*Leonurus heterophyllus*	Anti-inflammation in rat model and decreasing secretion of TNF-*α*, IL-6, and IL-8 in mast cells [45, 46] Anti-inflammation by inhibiting nuclear transcription factor kappa B pathway in human umbilical vein endothelial cells [47]

*Salvia miltiorrhiza*	Immunomodulation, decreasing peritoneal IL-18 and TNF-*α*, but elevating IL-13 in rat endometriosis model [48, 49]

*Taraxacum mongolicum*	Anti-inflammation by decreasing expression of TNF-*β* in lipopolysaccharide-induced human bronchial epithelial cells [50]

*Sparganium stoloniferum*	Induction of apoptosis by arresting cell cycles in neuroblastoma cells [51] Anti-inflammation by decreasing IL-1 and monocyte chemoattractant protein-1 in human umbilical vein endothelial cells [52]

## Abbreviations

ARM: Association rule mining
CHM: Chinese herbal medicine
HF: Herbal formula
ICD-9-CM: International Classification of Diseases, Ninth Revision, Clinical Modification
NHI: National Health Insurance
NHIRD: National Health Insurance Research Database
SH: Single herb
SNA: Social network analysis
TCM: Traditional Chinese medicine
GZFLW: Gui-Zhi-Fu-Ling-Wan
TNF-*α*: Tumor necrosis factor-alpha (TNF-*α*).
